# Structural Instability-Enabled Mechanical Sensors Using Fiber Bragg Grating

**DOI:** 10.3390/ma13112599

**Published:** 2020-06-07

**Authors:** Pengcheng Jiao, Yiwei Xie, Shengnan Wu, Xinyu Liu

**Affiliations:** 1 Institute of Port, Coastal and Offshore Engineering, Ocean College, Zhejiang University, Zhoushan 316021, China; pjiao@zju.edu.cn; 2 Engineering Research Center of Oceanic Sensing Technology and Equipment, Zhejiang University, Hangzhou 310058, China; 3 Centre for Optical and Electromagnetic Research, State Key Laboratory for Modern Optical Instrumentation, Zhejiang Provincial Key Laboratory for Sensing Technologies, Zhejiang University, Hangzhou 310058, China; 3140100676@zju.edu.cn; 4 Centre for Optical and Electromagnetic Research, National Engineering Research Center for Optical Instruments, Zhejiang Provincial Key Laboratory for Sensing Technologies, Zhejiang University, Hangzhou 310058, China; wushengnan@zju.edu.cn

**Keywords:** structural instability, mechanical sensors, fiber Bragg grating (FBG), pattern recognition, structural health monitoring (SHM)

## Abstract

Structural health monitoring (SHM) has been extensively used in civil infrastructures to assess structural condition and situation. Here, we develop a novel type of mechanical sensing technique using the structural instability of cylindrical cells detected by fiber Bragg grating (FBG). The cylinders are fabricated using a 3D printing technique, which are coiled by the FBG wires to detect the transverse deformation. Structural instability under axial compression is obtained in the experiments and the force–displacement relations are validated by the numerical simulations with satisfactory agreements. The wavelength variation of the FBG, caused by the structural instability, is observed and compared with the predefined threshold. Defining the variation larger than the threshold as “1” and smaller as “0”, the pattern recognition algorithm is used to convert the FBG results into binary data, which can, therefore, be analyzed to indicate the structural conditions. In the end, we envision the potential applications of the reported sensing technique, such as wireless sensors for structural health monitoring (SHM) in civil infrastructures.

## 1. Introduction

Structural health monitoring (SHM) has been extensively used in civil infrastructures to assess structural condition and situation, which requires autonomous, continuous, cost-effective, and reliable detection of structure performance. Damage detection is critical regarding SHM, and therefore various techniques have been developed to improve accuracy and efficiency, so as to detect damage at the early stage and prevent potential structural failure. Many sensing methods and mechanisms have been developed to investigate material and structural failures based on structural deformation (i.e., mechanical strain) [1,2]. Strain refers to the amount of deformation structures suffer due to internal or external excitations, and therefore strain sensing is the technique to detect and reflect the changes of the structure behavior. Traditional strain sensors (e.g., piezoresistive strain gauges) are typically based on electrical devices such as piezoresistors (devices that exhibit changes in resistance under strain changes) [3], and metal wire-based strain gauges or foil strain gauges [4]. However, those resistances typically result in complex and bulky measuring data. As a consequence, research efforts have been dedicated to developing new detecting techniques with good efficiency and accuracy.

Here, we develop a novel type of mechanical sensing technique that makes use of the structural instability of cylindrical cells. Fiber Bragg grating (FBG) is deployed to measure the structural response of the cylinders. Compared with the conventional electrical sensors, the FBG technique has various inherent advantages, such as non-conductivity, resistance to corrosion, and immunity to electromagnetic interference [5,6,7,8,9,10]. FBG has been reported with promising sensitivity and accuracy for deformation measurement [11,12,13]. FBG has been applied in many sensing applications, such as temperature sensors [14,15], acoustic sensors [16], and stress [17] or strain [18,19,20] sensors. In this study, the FBG observations are compared with predefined thresholds to convert the detected results into binary data. Analyzing data with the help of a pattern recognition (PR) algorithm, the mechanical response of the targeted structures can be detected. The PR method is a typical machine learning (ML) technique in artificial intelligence (AI), which has been extensively used to analyze image data and observe structural changes in SHM [21]. Taking advantage of FBG in detecting the structural instability (i.e., postbuckling) response, the detection data of the reported mechanical sensors are significantly reduced compared with time-dependent sensors. In particular, the predefined threshold is selected such that only a postbuckling response (i.e., tension- or compression-induced cylinder deformation) more critical than the threshold is recorded and converted into the binary data. As a consequence, the reported mechanical sensors are only triggered by a deformation larger than the threshold. The rest of the paper is presented as: Section 2 introduces the paradigm and design principle of the powerless mechanical sensors. Section 3 presents the experimental and numerical studies of the instability of cylindrical cells. Section 4 demonstrates the pattern recognition algorithm used to analyze the binary data detected by FBG. Section 5 envisions potential applications for the powerless mechanical sensors in SHM.

## 2. Design Principle and Paradigm

This section demonstrates the design principle of the mechanical sensor, while indicating the paradigm of the FBG for converting structural instability into binary data. PR is used to analyze the data and detect the damage of structures.

### 2.1. Design Principle of Powerless Mechanical Sensors Using Structural Instability

**Figure 1:**
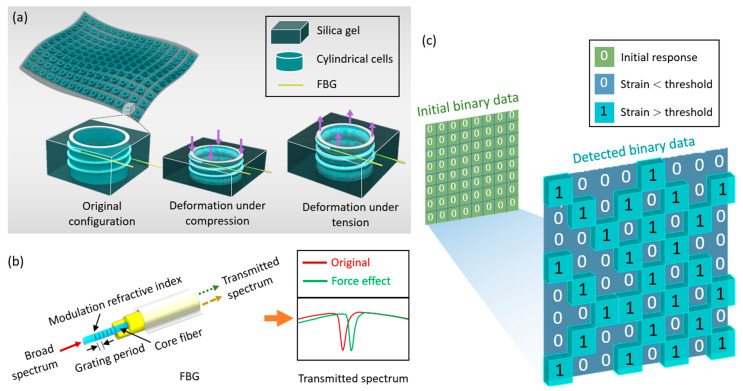
Design principle of the mechanical sensors. (**a**) Illustration of powerless mechanical sensors and configuration changes from original to deformed under external excitations. (**b**) Principle of fiber Bragg grating (FBG) under the influences of external force. (**c**) Binary data (i.e., detected response) converted from the structural instability, which indicate the deformation of the cylindrical cells larger than the predefined thresholds.

Figure 1 demonstrates the design principle of the mechanical sensors using FBG-enabled pattern recognition to consider structural instability. Figure 1a illustrates the original and deformed configurations of the cylindrical cells in the mechanical sensors under external excitations, such as strain fluctuations. The cylindrical cells in the mechanical sensors are coiled by FBG, such that the structural configurations can be accurately measured. Figure 1b demonstrates the principle of FBG used to measure the structural instability of the cylinders. FBG is formed by the periodic change of the refractive index of the fiber core in the direction of the propagation of optical radiation. Full-spectrum light is transmitted into one end of the FBG core, which is separated into two light signals at the location of the Bragg grating. External mechanical variations lead to an associated shift of the central Bragg wavelength of the optical fiber sensor, as indicated by the reflected light or transmitted light in the figure. In this condition, the FBG acts as a spectral filter that reflects particular wavelengths of light near the Bragg resonance wavelength and releases the rest. The spectrum of the FBG is shown by the red line, while the transmitted spectrum that resulted in force is given in green. According to the spectral features of FBG, the deformations of the cylindrical cells in the mechanical sensors can be captured.

Defining certain configuration thresholds, the deformations of the cylinders smaller than the threshold are referred to as the signal of “0”, while the deformations larger than the threshold are “1”. As a consequence, the structural response of the 8 × 8-unit mechanical sensor is converted into binary data. Figure 1c displays the binary data matrices before and after the deformations.

In particular, the 8 × 8 “0” data matrix represents the initial mechanical response of the structures. Detecting structural strains using FBG at an arbitrary time step $t_{i}$ and comparing it with the threshold, those cylinders deformed more than the threshold are changed to “1” and the smaller ones are maintained as “0”. When the time step is increased to $t_{i+1}$, the binary data matrix is updated accordingly. A PR algorithm is then used to compare the detected data matrices, and the pattern differences are analyzed to detect the structural damage under the external excitations (e.g., force in Figure 1a). Compared with the time-dependent sensors, the advantage of the reported mechanical sensors is the significant reduction in the amount of detection data. In particular, the predefined threshold is selected such that only a postbuckling response (i.e., tension- or compression-induced cylinder deformation) more critical than the threshold is recorded and converted into the binary data. As a consequence, the reported mechanical sensors are only triggered by a deformation larger than the threshold, which does not particularly distinguish tension from compression.

### 2.2. Paradigm of FBG in Detecting Structural Instability

The Bragg resonant wavelength is the dominant factor in FBG, which can be expressed as [22]:

(1) $$\lambda_{\mathrm{FBG}}=2n_{\mathrm{eff}}\Lambda$$

where $\lambda_{\mathrm{FBG}}$, $n_{\mathrm{eff}}$, and Λ refer to the Bragg resonant wavelength, refractive index, and grating period, respectively. Note that the Bragg resonant wavelength is determined by various factors applied on FBG (e.g., mechanical deformation fluctuation), which significantly affect the refractive index or grating period. In return, the changes of the Bragg resonant wavelength accurately reflect the environment variation for FBG. Coiling FBG around the cylinders (Figure 1b), the deformations lead to spectra transmission.

The Bragg resonant wavelength is typically affected by changes in mechanical conditions and ambient temperature. Therefore, it is desirable to distinguish the thermal and mechanical influences on the spectrum, so as to identify the state of the cylinders. Considering the length extension and cross-sectional shape changes of the cylinders, while omitting the thermal influence following the stress-strain relationship, the wavelength shifting of the FBG caused by the changes in the strain or temperature is given by [23,24,25]:

(2) $$∆\lambda_{\mathrm{FBG}}=2\left(\Lambda \frac{\partial n_{\mathrm{eff}}}{\partial l}+n_{\mathrm{eff}}\frac{\partial \Lambda }{\partial l}\right)\Delta l+2\left(\Lambda \frac{\partial n_{\mathrm{eff}}}{\partial T}+n_{\mathrm{eff}}\frac{\partial \Lambda }{\partial T}\right)\Delta T$$

where Δl and ΔT are the changes in the FBG’s length and temperature. Considering the length extension or cross-section shape changes, while omitting the thermal influence, the wavelength response of FBG at any point can be expressed as:

(3) $$\{\begin{array}{c} {\left(∆n_{\mathrm{eff}}\right)}_{x}=-\frac{n_{\mathrm{eff}}{}^{3}}{2E}\left[\left(p_{11}-2\nu p_{12}\right)\sigma_{x}+\left[\left(1-\nu \right)p_{12}-\nu p_{11}\right]\left(\sigma_{y}+\sigma_{z}\right)\right]\\ {\left(∆n_{\mathrm{eff}}\right)}_{y}=-\frac{n_{\mathrm{eff}}{}^{3}}{2E}\left[\left(p_{11}-2\nu p_{12}\right)\sigma_{y}+\left[\left(1-\nu \right)p_{12}-\nu p_{11}\right]\left(\sigma_{x}+\sigma_{z}\right)\right] \end{array}$$

where *E* and ν are, respectively, the Young’s modulus and Poisson coefficient of the optical fiber, $p_{11}$ and $p_{12}$ are the strain-optic coefficients, and $\;n_{\mathrm{eff}}$ is the average refractive index along the two orthogonal axes of the fiber. $\sigma_{x}$, $\sigma_{y}$, and $\sigma_{z}$ are the stress components of the FBG in the x, y, and z principal directions, respectively. Using the Hooke elasticity relationship, the Bragg reflection wavelength at any point on the disturbed FBG can be written as [26]:

(4) $$\left\{ \begin{array}{l} ∆\lambda_{x}=\lambda_{x}\left[-\frac{n_{\mathrm{eff}}{}^{2}}{2E}\times \left[\left(p_{11}-2\nu p_{12}\right)\sigma_{x}+\left[\left(1-\nu \right)p_{12}-\nu p_{11}\right]\times \left(\sigma_{y}+\sigma_{z}\right)\right]+\frac{1}{E}\times \left[\sigma_{z}-\nu \left(\sigma_{x}+\sigma_{y}\right)\right]\right]\\ ∆\lambda_{y}=\lambda_{y}\left[-\frac{n_{\mathrm{eff}}{}^{2}}{2E}\times \left[\left(p_{11}-2\nu p_{12}\right)\sigma_{y}+\left[\left(1-\nu \right)p_{12}-\nu p_{11}\right]\times \left(\sigma_{x}+\sigma_{z}\right)\right]+\frac{1}{E}\times \left[\sigma_{z}-\nu \left(\sigma_{x}+\sigma_{y}\right)\right]\right] \end{array}\right.$$

where *∆λx* and *∆λy* are the wavelength shifting in the fast and slow axes, and *λx* and *λy* are the initial wavelengths of the peaks corresponding to the two polarization modes, respectively.

Assuming the ambient temperature is constant, the Bragg wavelengths of the two polarization modes provide different shifts in the *x* and *y* axes (i.e., amplitude and period) under the strain changes (the green line in Figure 1b). On the contrary, when the force is constant, temperature fluctuation only causes wavelength shifting in the x-axis of the transmitted spectrum due to the thermo-optic effect and the thermal expansion effect, as shown by the yellow line in Figure 1b. Therefore, the tension- and compression-induced strain can be obtained based on the difference in Bragg wavelengths between the two polarization modes. However, since single mode fiber has ultra-low birefringence, radial strain can be ignored in our model, namely, the theoretical force analysis toward FBG can be simplified to be affected only by axial strain. The simplified equation can be written as [25,27]:

(5) $$∆\lambda_{\mathrm{FBG}}=\lambda_{\mathrm{FBG}}\times \left\{1-\frac{n_{\mathrm{eff}}{}^{2}}{2}\times \left[p_{12}-\nu \left(p_{11}+p_{12}\right)\right]\right\}\times \varepsilon_{z}\;,$$

where $\varepsilon_{z}$ is the longitudinal strain. It is worthwhile to point out that the temperature changes can be compensated for by adding another FBG to only detect the temperature or placing the device under test in a temperature-controlled environment.

## 3. Experiments and Numerical Simulations on the Structural Instability of Cylindrical Cells

In this section, the cylinders were fabricated using the 3D printing technique, which were coiled by the FBG wires. Experiments were then carried out on the structural instability of the FBG cylinders, and the experimental results were validated with numerical simulations. The FBG results were obtained in wavelength to detect the deformation of the cylinders in the experiments.

### 3.1. Fabrication of FBG Cylindrical Cells

**Figure 2:**
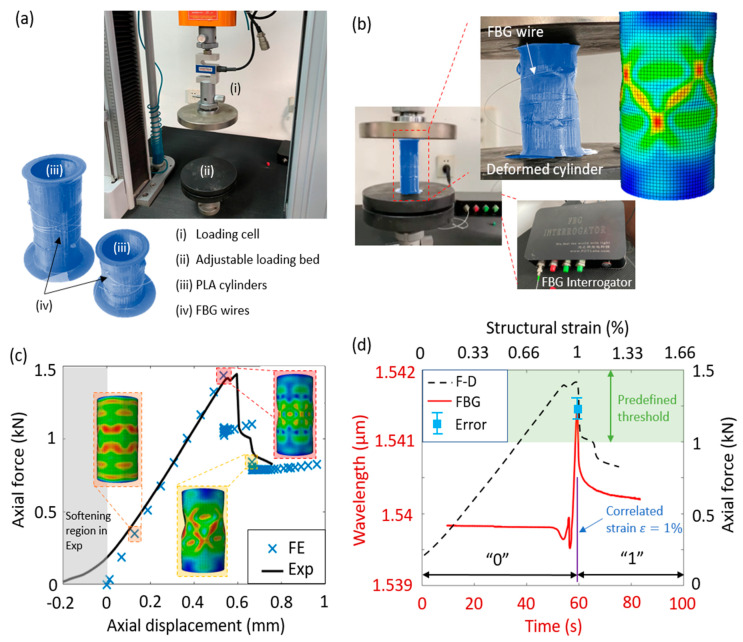
Experiments and numerical simulations of the FBG cylinders subjected to external loading. (**a**) 3D printed cylinders coiled by FBG wires and the loading machine in the experiments. (**b**) Experimental setup, testing results, and numerical simulations of the deformation configurations of the cylindrical samples. (**c**) Comparison of the force–displacement relations between the experimental and numerical results and the deformed shapes at the limit states. (**d**) FBG detection and force–displacement relation of the cylinder and the conversion of structural instability into binary data, using the predefined threshold of wavelength of 1.541 µm.

The cylindrical cells in the mechanical sensors were manufactured using a polymer-based 3D printing device [28] with polylactic acid (PLA) filaments. In particular, PLA filaments were heated and squeezed out through the nozzle, and were 0.4 mm in diameter. The filaments were then rapidly cooled down once they were placed on the structuring cylindrical samples. The entire process was repeated, such that the cylinders were fabricated layer by layer. The top and bottom ends of the cylinders were fabricated with a greater thickness, such that local damages could be prevented under uniform axial compression. The FBG wires, of about 5 mm length and 90% reflectivity, were inscribed on a hydrogen-loaded SMF (SM28, Corning, NY, US) by using a 193 nm ArF excimer laser (BraggStar S-Industrial [29], Coherent Inc., Santa Clara, CA, US) with a phase mask method. Figure 2a displays the 3D printed cylinders coiled by the FBG wires and the Gotech loading machine. In particular, ultra-violet glue (Model NOA65, Norland Ltd., CRANBURY, NJ, US) was used to bond the FBG wires to the cylinders. The fabrication process of the FBG wires was based on the commercial phase mask method, which is suitable for mass production [30]. In addition, the packaging can be reproduced by using mature commercial glue, such as Norland NOA65 glue or EPOXY-353ND glue. The FBG wires were covered over all the grating area by UV glue. Note that only one FBG was designed to detect the postbuckling response of each cylinder under axial tension and compression. The certain threshold of the postbuckling response is predefined (Figure 2d), such that the FBG only monitors a deformation more severe than the threshold, while the less critical events are neglected to significantly reduce the amount of the detection data. The loading machine comprised the loading cell and adjustable loading bed. To avoid the imperfection of the initial tilt, the loading bed was designed with a semi-sphere placed between two flat plates.

### 3.2. Experiments and Numerical Simulations

The loading machine was used to test the structural deformations of the cylinders subjected to axial compression. Quasi-static loading conditions were applied, such that the FBG cylinders cannot be destroyed in the loading–unloading process. In particular, the axial compression of 0.8 mm was applied to ensure the functionality of the FBG wires under deformation. Figure 2b presents the experimental setup, and the comparison of the deformation configurations between the experimental and numerical results. In the experiments, the sample was placed in the center of the loading bed, and the FBG interrogator was used to obtain the detected wavelength. In the numerical simulations, the finite element (FE) model was developed in Abaqus v16.1. The cylinders were simulated using the shell elements (S4R). Buckling and postbuckling analyses were conducted to obtain the postbuckling response of the cylinders subjected to axial compression. In particular, the buckling analysis was linear perturbation/buckle, and the postbuckling analysis was dynamic implicit with Nlgeom. The excitation was applied to the top edge of the cylinders as axial displacement. The geometric and material properties, element type and size, and loading conditions are summarized in Table 1.

Figure 2c shows the comparison of the force–displacement relations between the experimental and numerical results and the deformed cylinder configurations at the limit states. Shifting to exclude the initial softening, the experimental results are observed having the same stiffness and amplitude. The deformed configurations are presented when the cylinder starts deforming, as well as before and after the structural instability. It can be seen that satisfactory agreements are obtained between the numerical and experimental results.

### 3.3. FBG Detection of the Structural Instability of the Cylindrical Cells

Figure 2d illustrates the FBG sensing results of the deformed cylinder samples. We used FBG to obtain the variations of the wavelength before and after the structural instability of the cylinder. We define the variation threshold of the wavelength as 1.541 µm, which leads to the strain threshold of ε=1%, and therefore the conditions before the threshold (i.e., ε<1%) can be converted into the binary data “0”, while those after the threshold are “1”. It is worthwhile to mention that the structural instability (strain threshold) can be programmed. Designing the FBG cylinders with different thresholds and assembled into sets, the obtained binary data can be used to detect the conditions of structures, as demonstrated in Figure 1c. In addition, three different cylindrical samples were tested twice under the cyclic loading to ensure the repeatability of the FBG sensors. Since the mechanical strains considered in this study are relatively small, the detected results are repeatable and comparable. The errors in the estimation of the thresholds of the cylinders are approximately 5% of the peak value, as shown with the error bar in Figure 2d.

## 4. Pattern Recognition Analysis and Discussion on Potential Applications

### 4.1. Pattern Recognition Algorithm

Next, we use the damage detection concept to analyze the FBG detected binary data from the mechanical sensors. The binary data generated from the response of structures are analyzed into patterns. The detection procedures can be summarized as using the validated simulation results of structural instability as feature vectors to obtain binary data to analyze. Afterward, the recognized patterns are incorporated with conditional chains to identify damage. In particular, pattern variations are anticipated when damage happens to the structures, which are then used to assess structural conditions.

**Figure 3:**
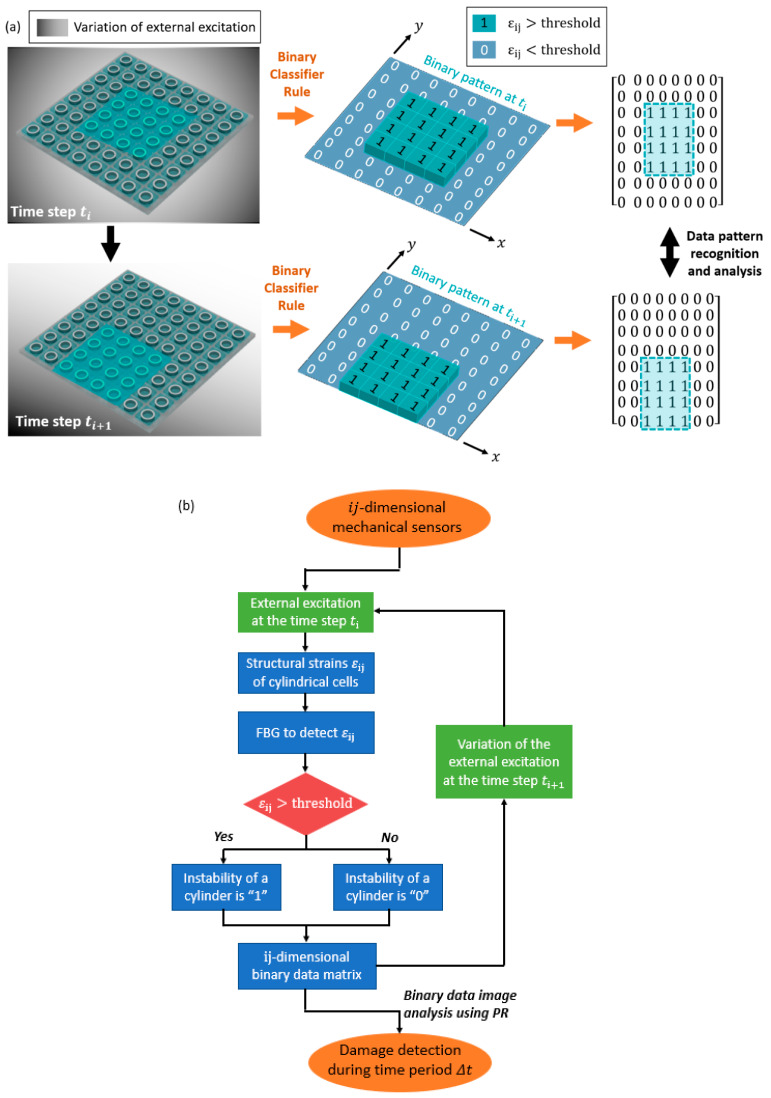
Pattern recognition scheme for the FBG-enabled binary data from the mechanical sensors. (**a**) Schematic illustration of the pattern scheme that analyzes the binary data detected from the FBG. (**b**) A flowchart that indicates the procedures of the binary classifier rules and data analysis.

Figure 3a presents the schematic illustration of the pattern scheme of analyzing the FBG binary data of the mechanical sensors. Figure 3b displays the flowchart that details the developed damage detection approach. In this study, we use pattern recognition (PR) as the image processing method to recognize and analyze the binary data generated from the FBG wires, detecting the condition of structures from normalness to irregularity [31,32,33]. Pattern deviation is used to analyze the binary data with respect to each other in the PR analysis. In particular, the binary data detected from the structural response using FBG were considered as image patterns. According to the PR scheme, each pattern (i.e., binary data detected at a certain time $t_{i}$) is addressed as a data matrix and characterized with image features based on binary values. Note that the dimension of the binary data is dependent on the number and distribution of the FBG cylinders in the mechanical sensors. If a mechanical sensor consists of n=i×j cylindrical cells, each binary datum (i.e., pattern matrix) can be represented with n features. Therefore, data classifiers are used to search for the shifting of the patterns in the sensor. When the detected binary data are recorded at the time step of $t_{i+1}$, the extracted features of the binary data are compared with the features from the previously recorded pattern at the time step of $t_{i}$. If all the n features are identical between the time steps of $t_{i}$ and $t_{i+1}$, the binary data are characterized to be in the same class.

Otherwise, the data at $t_{i+1}$ is classified as a new class. Following the pattern classifier, FBG-enabled binary data can be used to filter the ambient temperature or load excitation variations that are larger than the predefined thresholds.

### 4.2. Potential Applications of the Mechanical Sensors for Structural Health Monitoring (SHM)

**Figure 4:**
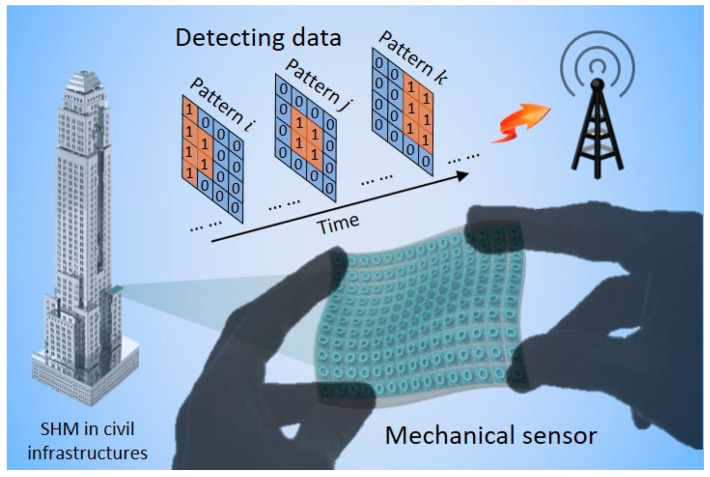
Potential application of the mechanical sensors for measuring structural conditions for SHM in civil infrastructures.

Here, the wireless mechanical sensors are outlooked in civil infrastructures for structural health monitoring (SHM). Figure 4 illustrates the application of the mechanical sensors for SHM in civil infrastructures. The mechanical sensors are assembled onto a platform, which are embedded in the targeted structures. The light source and optical interrogator are designed into the supply device box. The detecting signals are wirelessly sent to signal transmission devices, which are delivered to central computers via the internet. As a consequence, the mechanical sensors are capable of measuring the structural conditions and the results can be wirelessly sent to smartphones for analysis.

## 5. Conclusions

In this study, we developed mechanical sensors based on the structural instability of cylindrical cells detected by fiber Bragg grating (FBG). The cylinders were fabricated using 3D printing and the FBG wires were coiled to detect transverse deformation. Experiments were conducted to obtain structural instability under axial compression, and the experimental results (i.e., force–displacement relations) were validated by numerical simulations with satisfactory agreements. The wavelength variation of the FBG was observed due to structural instability, and the results were compared with the predefined threshold. The pattern recognition algorithm was used to convert the FBG results into binary data by defining a variation larger than the threshold as “1” and smaller as “0”. The binary data was analyzed to indicate the structural conditions, and the reported technique was envisioned as wireless sensors for SHM in civil infrastructures.

## Figures and Tables

**Table 1:** Geometric and material properties, element type, and size in finite element (FE) modeling, and loading conditions.

		Cylinders (Polylactic Acid PLA)	Fiber Bragg Grating (FBG)
Mater.	Density (g/cm^3^)	1.24	2.20
	Young’s modulus (GPa)	3.47	73.02
	Elongation at break (%)	5.2	2
	Tensile modulus	1.34	--
Geo.	Length (mm)	50, 60, 70	5
	Diameter (mm)	30	0.125
	Thickness (mm)	0.4	--
FE	Element type	S4R	--
	Element size (mm)	3	--
Load	Amplitude (mm)	0.8	
	Time (s)	100	
